# Barotrauma and delayed perforation upon endoscopic laser ablation of the duodenal mucosa for type 2 diabetes: a case report

**DOI:** 10.1055/a-2769-7106

**Published:** 2025-12-17

**Authors:** Kim van den Hoek, Celine B. E. Busch, Sabrine Q. Kol, Mark I. van Berge Henegouwen, Annieke C. G. van Baar

**Affiliations:** 1 1209 Department of Gastroenterology and Hepatology, Amsterdam University Medical Center, University of Amsterdam, Amsterdam, The Netherlands; 2 571165 Amsterdam Gastroenterology Endocrinology Metabolism, Research Institute, Amsterdam, The Netherlands; 3 522567 Department of Radiology and Nuclear Medicine, Amsterdam University Medical Center, Amsterdam, The Netherlands; 4 Department of Surgery, Amsterdam University Medical Center, University of Amsterdam, Amsterdam, The Netherlands; 5 571143 Cancer Center Amsterdam, Cancer Treatment and Quality of Life, Amsterdam, The Netherlands

Endoscopic duodenal ablation has emerged as a minimally invasive technique to improve glycemic control in type 2 diabetes. The Digma System (Digma Medical Ltd, Givat Shmuel, Israel) delivers circumferential submucosal laser ablation via a balloon catheter ( Fig. 1 ). A first-in-human study reported the procedure to be safe, without procedure-related complications 1 . We report a case of severe respiratory insufficiency with extensive air dissemination following ablation with the Digma System in a 72-year-old woman enrolled in a clinical study.

**Fig. 1:**
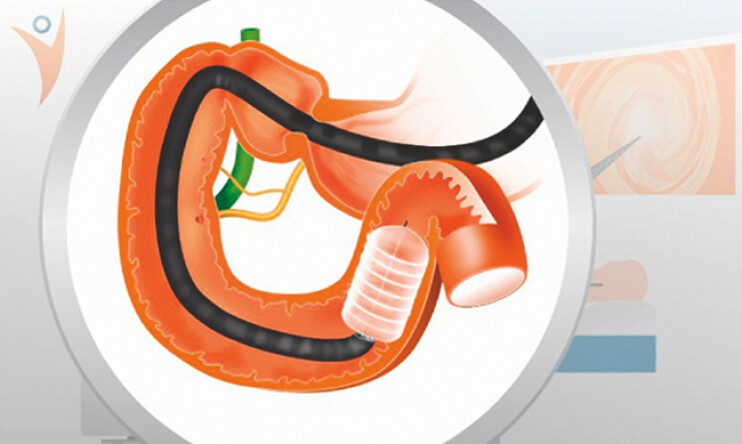
Duodenal submucosal laser ablation with the Digma System via a through the scope balloon catheter.

No abnormalities were found during screening endoscopy ( Video 1 ). During ablations in the descending duodenum, the patient developed inspiratory stridor and desaturation to 40%. The endoscope was removed and endotracheal intubation was performed.

An endoscopic view through the balloon catheter showing circumferential duodenal laser ablation.Video 1

Computed tomography (CT) showed free air in multiple anatomical compartments ( Fig. 2 ). No visible perforation was identified on CT or during diagnostic laparoscopy ( Fig. 3 ), suggesting barotrauma without overt perforation as the most likely cause.

**Fig. 2:**
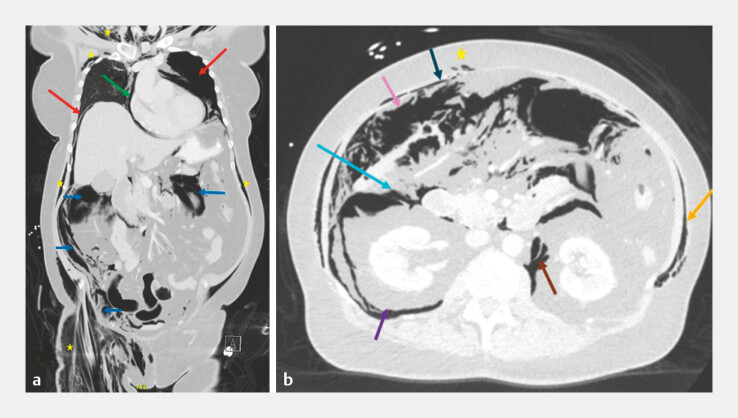
**a** A coronal CT scan of the chest and abdomen showing bilateral pneumothorax (red arrows), diffuse subcutaneous emphysema (yellow stars), pneumopericardium (green arrow), and intra-abdominal air (blue arrows). **b** An axial CT scan of the abdomen showing free air in different anatomical compartments: subcutaneous tissues (yellow star), between abdominal wall muscles (orange arrow), preperitoneal space (dark blue arrow), anterior pararenal space (light blue arrow), peritoneal cavity (pink arrow), retroperitoneal space (brown arrow), and posterior pararenal space (purple arrow). CT, computed tomography.

**Fig. 3:**
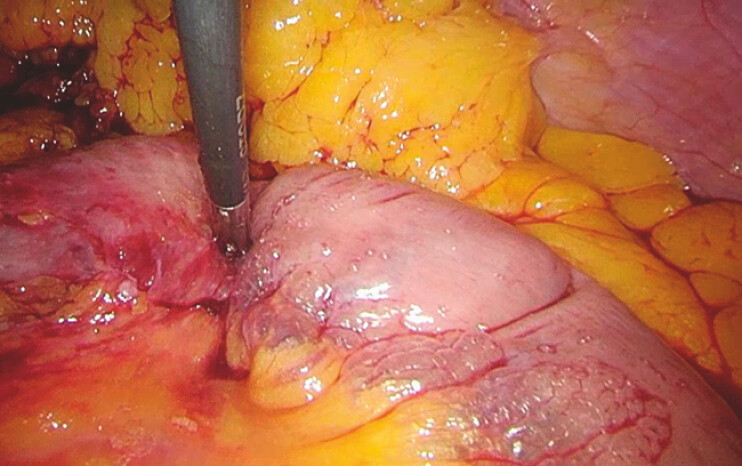
Diagnostic laparoscopy showing intraperitoneal air within the mesentery of the proximal small bowel, near the ligament of Treitz, visible as small gas collections.

The patient was treated conservatively in the intensive care unit. A CT scan with oral contrast the next day showed no contrast leakage. On day 3, the patient was extubated and transferred to the gastrointestinal ward. On day 14, CT imaging revealed a duodenal perforation near the ligament of Treitz, again managed conservatively ( Fig. 4 ). Patient was discharged after 41 days. Follow-up endoscopy showed a healed ulcer and concentric ablation rings.

**Fig. 4:**
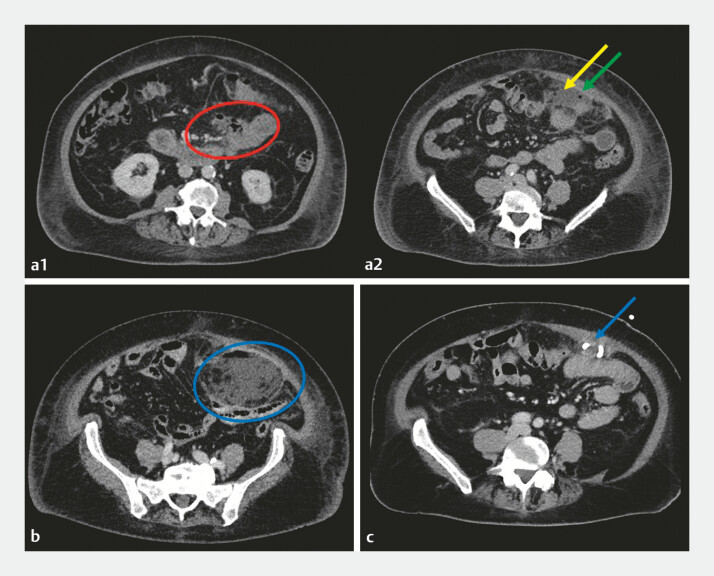
Axial CT scans of the abdomen. **a1, a2** Day 14 post-procedure. **a1** Defect of the medial duodenal wall at the level of the ligament of Treitz, with associated free fluid and extraluminal air, findings consistent with a perforation (red circle). **a2** Non-encapsulated fluid with associated infiltration of the surrounding intra-abdominal fat (yellow arrow) accompanied by small extraluminal air locules (green arrow) in the left anterior abdomen. **b** Day 25 post-procedure: enlarging fluid collection with an air-fluid level (blue circle), continuous with the duodenal perforation (not shown). **c** Day 39 post-procedure: 11 days after placement of a percutaneous pigtail drain (blue circle) shows a marked reduction in fluid collection. CT, computed tomography.

Root cause analysis identified multiple micro-perforations in the ablation balloon and compensatory over-insufflation (>11 L of room air). Barotrauma likely resulted from combined thermal injury and room-air leakage through these micro-defects. Endoscope or patient movement affected laser rotation, leading to longer and deeper ablations. System modifications now include CO _2_ insufflation, air–volume limits, and endoscope–catheter fixation to prevent inadvertent fiber motion. This case underscores the need for rigorous safety monitoring when introducing novel endoscopic techniques, even when preclinical data suggest safety.

Endoscopy_UCTN_Code_CPL_1AH_2AJ

## Notes

Endoscopy E-Videos https://eref.thieme.de/e-videos
*E-Videos* is an open access online section of the journal *Endoscopy* , reporting on interesting cases and new techniques in gastroenterological endoscopy. All papers include a high-quality video and are published with a Creative Commons CC-BY license. Endoscopy E-Videos qualify for HINARI discounts and waivers and eligibility is automatically checked during the submission process. We grant 100% waivers to articles whose corresponding authors are based in Group A countries and 50% waivers to those who are based in Group B countries as classified by Research4Life (see: https://www.research4life.org/access/eligibility/ ). This section has its own submission website at https://mc.manuscriptcentral.com/e-videos .
